# Smoked and Fermented Bushmeat (Mpunam) Products: Risk Assessment of Polycyclic Aromatic Hydrocarbons (PAHs) Resulting From Processing

**DOI:** 10.1155/2024/5514988

**Published:** 2024-10-16

**Authors:** A. S. Amponsah, H. E. Lutterodt, G. M. Ankar-Brewoo, I. W. Ofosu

**Affiliations:** ^1^Department of Hospitality and Tourism, Sunyani Technical University, Sunyani, Ghana; ^2^Food Systems Chemistry, Toxicology, and Risks Studies, Department of Food Science and Technology, College of Science, Kwame Nkrumah University of Science and Technology (KNUST), Kumasi, Ghana

**Keywords:** bushmeat, excess lifetime cancer risk, margin of exposure, PAH, smoked

## Abstract

The polycyclic aromatic hydrocarbons (PAHs) congener concentrations and risk upon human exposure to smoked bushmeat products were analyzed. GC/MS MRM and QuEChERS methods were used for the analysis. This work has become necessary due to the need for more information concerning the quantitative determination of these compounds and their health risk assessment. The 16 PAH congeners identified were acenaphthylene (ACA), naphthalene (NAP), acenaphthene (ACE), fluorene (FLU), anthracene (ANT), phenanthrene (PHE), fluoranthene (FLT), pyrene (PYR), benzo[b]fluoranthene (BBF), benzo[k]fluoranthene (BKF), benzo[a]anthracene (BAA), chrysene (CHR), indeno(1,2,3-cd)pyrene (IND), dibenzo(a,h)anthracene (DAA), benzo(g,h,i)pyrene (BGP), and benzo[a]pyrene (BAP). At the 5% and 95% daily intake levels, BAP was at 3.34 and 17.39 *μ*g/kg(bw)/day, *Σ*PAH4 was at 25.11 and 109.15 *μ*g/kg(bw)/day, and *Σ*PAH8 was at 55.76 and 236.68 *μ*g/kg(bw)/day, respectively. BAP, *Σ*PAH4, and *Σ*PAH8 concentration exceeded the European Union limits, as BAP concentration was as low as 6.09 *μ*g/kg and as high as 34.19. The exposure values were significantly high. Specifically, the margin of exposure for BAP was as low as 2.09 × 10^−2^; for *Σ*PAH4, it was 1.36 × 10^−−2^; and for *Σ*PAH8, it was 1.95 × 10^−2^ all at the 95% level. These figures are substantially lower than the benchmark of 10,000, indicating a higher ILTCR. Furthermore, the ILTCR ranged from a minimum of 47.77 to a maximum of 248.53 at the 5% and 95% levels, respectively. This study makes smoked bushmeat a public health concern because the higher figures obtained indicate higher carcinogenicity upon consumption.

## 1. Introduction

The bushmeat trade primarily sources animal protein as an income–earning commodity for markets across African tropical forest regions. However, concerns about their safety could attract more research attention. Bushmeat, sometimes known as game meat, is described as meat obtained from wild animals or a word referring to using wild animals for food, such as cane rats and gorillas. The availability of substitute proteins, affluence, and price impact how much bushmeat is consumed [1].

Studies have repeatedly demonstrated that bushmeat is sensitive to its price and consumer wealth. In line with economic theory, when bushmeat's price rises, its consumption declines, and this effect is mediated by changes in wealth [2]. African bushmeat from the Savanna is typically inexpensive [3], thus influencing its high consumption.

However, in Ghana, the harvesting and trading of bushmeat is permitted for most of the species. The bushmeat trade in Ghana is governed by the Wild Animal Preservation Act 1961 (Act 43), which controls the hunting of protected species and sites during the hunting season. The trade is also regulated by the issuance of licenses with three independent institutions: the police, who are in charge of shotgun licenses; the wildlife department for hunting licenses; and finally, the District Assemblies for trade licenses [4].

The bushmeat on the market is either fresh, smoke-fermented (dry) or fresh-fermented. The smoking of bushmeat is aimed at preserving it to survive the distribution chain. Other reasons are the preference for characteristic flavour, colour, compactness of the fermented product, longer shelf life, and ease of transportation [5]. Judging from the habitual smoking habits of hunters, polycyclic aromatic hydrocarbons (PAHs) are no doubt a source of contaminants in bushmeat products. This results from incomplete burning of organic substances, wood smoke, waste incineration, and other anthropometric processes that produce PAH and pollute the environment. Human exposure to PAH is even worsened by natural emissions such as those from volcanoes and forest fires [6].

PAHs are gaining public attention because of the health risks associated with them. Studies have shown them to have adverse effects on biological systems bordering on mutagenicity, carcinogenicity, teratogenicity, and immunotoxicogenicity [7]. These aromatic molecules, also known as PAHs, are composed of multiple fused benzene rings, forming their distinctive structural framework. Pyrogenic sources of PAHs often have heavy molecular weights greater than six rings, whereas petrogenic sources consist of lighter molecular weights, often with less than six rings [8]. They are also classified into alternants, consisting of fused six-member benzene rings, and nonalternants, which are mixed of six and five members.

To assess food safety, international organizations regulate PAH levels [9]. Among the hundreds of PAHs, 16 are prioritized due to their health risks. These include acenaphthylene (ACA), naphthalene (NAP), acenaphthene (ACE), fluorene (FLU), anthracene (ANT), phenanthrene (PHE), fluoranthene (FLT), pyrene (PYR), benzo[b]fluoranthene (BBF), benzo[k]fluoranthene (BKF), benzo[a]anthracene (BAA), chrysene (CHR), indeno(1,2,3-cd)pyrene (IND), dibenzo(a,h)anthracene (DAA), benzo(g,h,i)pyrene (BGP), and benzo[a]pyrene (BAP).

Similarly, the European Food Safety Authority sees the sum of concentrations of four PAHs (*Σ*PAH4): BAP, CHR, BAA, and BBF as superior indicators of carcinogenicity, European Union Food Safety Authority (EFSA, 2008). Thus, comparing the sum of *Σ*PAH4 with those of the eight PAHs (∑PAH8) does not provide any additional value [10].

From such grouping, a compilation of EFSA considers ∑PAH4 not to exceed 12 *μ*g/kg [11], while the standard for BAP in meat products is set at 2 *μ*g/kg [12]. It has been established that dietary intake accounts for 90% of total PAH exposures [2], and their quantities are determined by the cooking techniques, variety of oil, temperature of wood, coal type, the extent of combustion, and the nature of the heat source [13]. Human exposure is of public health concern since PAH-specific bioaccumulation, toxicity, toxicokinetic, and toxicodynamic properties have been demonstrated to have adverse effects [14]. Indeed, major international bodies have classified PAHs into several groups depending on animal carcinogens, probably carcinogenic to humans or not classifiable as carcinogenic to humans [15].

PAHs require metabolic activation to enhance their properties as ultimate toxicants. This metabolic activation process is crucial for manifesting their toxic effects. Once in the liver, the cytochrome P450 enzymes mediate the oxidization of PAHs, thus initiating their bioactivation or detoxification pathways [16]. PAHs share a mechanism of carcinogenic action in humans and experimental animals, so a typical PAH such as BAP gets transformed into reactive metabolites such as BAP-7,8-dihydrodiol-9,10-epoxide. This metabolite can bind with the vulnerable nucleotide bases of the DNA to form adducts that manifest as mutations and eventually cause cancer [17]. However, the highly polar biomolecules in Phase II mediated by their enzymes, such as glutathione transferases or uridine 5-diphosphate-glucuronosyltransferase, contribute to solubilizing the BAP epoxides. The resulting soluble metabolites, BAP-dihydrodiols, BAP-diones, or their hydroxylated counterparts, are then excreted [18]. Literature is replete with information on PAHs from many sources. However, the presence of PAHs, especially in smoked meat products and, by extension, smoked bushmeat, has yet to receive much attention [12]. In this light, this study was aimed at characterizing the types of PAHs usually associated with bushmeat products, making it possible to quantify the exposures and the associated risks.

## 2. Materials and Methods

### 2.1. Materials

Four bushmeat samples (rabbit, rush rat, mona monkey, and grasscutter), which were all acquired through gunshot hunting, were purchased from hunters in Adentia, a village near the forest zone around Sunyani in the Bono Region of Ghana. The bushmeat was received fresh from hunters on arrival from the forest, where they spent 24–48 h hunting. After thorough inspection (evidence of gunshot, quality checks on fur to avoid purchasing meat kept for more than 24 h), the meat was accepted for further processing. The weight of each meat was measured, the fur was removed by smoking, and the meat was salted individually using 100% rock salt and kept for 24 h at room temperature before being smoked. In the smoking session, the smoking setup was adopted from a description by other authors for traditional meat processing in Ghana [19], where samples were laid out on a platform of wire mesh supported by a circular framework of a perforated metal drum measuring 0.857 m^2^. The fire was allowed to heat up from the base for about 4 h before the meat was placed on it for 8 h. The base of the drum from where the fire was made was stabilized with a pile of sand, while pieces of teak tree of an average length of 0.6 m and 0.05 m thick were used. An average temperature of 73°C was recorded during smoking using a Monotaro thermometer (CHE-TN430, Japan). The resultant bushmeat product was stored for 28 days in a jute sack at room temperature, within which samples were taken for analysis. Samples were placed in sterile plastic bags and immediately transferred into a refrigerated cold box (about 4°C) to the laboratory, where they were stored in freezers (−20°C).

#### 2.1.1. Chemicals and Reagents

Acetonitrile (HPLC grade, min. 99.9%) was purchased from Scharlab S.L. (Spain) and used without further filtration. The water used in all experiments was from a Milli-Q Reference Water Purification System with a resistivity of 18 M*Ω* cm. Helium (min. 99.9999%) and nitrogen (min. 99.996%) were both procured from Linde GmbH (Germany). QuEChERS extraction cartridge was purchased from Thermo Fisher Scientific Inc. (United States) and consisted of 50 mL centrifuge tubes containing 6 g anhydrous magnesium sulphate and 1.5 g sodium acetate. QuEChERS dSPE purification kits for fatty samples were purchased from Agilent Technologies Inc. (United States). They consisted of 15 mL centrifuge tubes containing 400 mg PSA (primary–secondary amine), 400 mg C18-silica, and 1200 mg anhydrous magnesium sulphate. A JTB-005 certified reference material from Agilent Technologies Inc. used for PAH chromatographic characterization and recovery determination consisted of 16 PAH congeners in acetonitrile, as presented in Table 1.

### 2.2. Methods

#### 2.2.1. Extraction Procedure

The extraction procedure was adopted from PAH analysis in fish by GC/MS using Agilent Bond Elut QuEChERS dSPE sample preparation and high-efficiency DB-5 ms Ultra Insert G.C. column [20], where 300 g of bushmeat samples were frozen at −80°C overnight after being chopped into smaller sizes. The samples were then grinded thoroughly to achieve a uniform mixture. Three grams of bushmeat was weighed into centrifuge tubes. Samples were spiked with an appropriate amount of PAH spiking solution to yield a sample concentration of 25, 250, and 500 ng/mL. Each sample had 12.0 mL of deionized water and 15 mL of ACN.

To aid extraction, two ceramic bars (p/n 5982-9313) were added to each sample. All samples were vortexed for 1 min. An original extraction salt packet was added to each centrifuge tube. The capped tubes were shaken on a Geno Grinder at 1500 rpm for 1 min and centrifuged at 4000 rpm for 5 min. Eight milliliters of the upper layer was transferred to the Agilent Bond Elut QuEChERS fatty sample dispersive tube. The tube was vortexed for 1 min and centrifuged at 4000 rpm for 5 min to complete the extraction process. The liquid from the dSPE tube was transferred to a G.C. vial and analyzed by SIM GC/MS using the chromatographic condition in Table 2.

#### 2.2.2. Risk Assessment

Generally speaking, a substantial number of PAH congeners were studied (*Σ*PAH4 and *Σ*PAH8) combined with all BAP, regarded as appropriate markers for PAH in food, and were identified in the bushmeat analyzed. Using the USEPA guidelines (Equation (1)), the chronic exposure was determined as follows:

(1) $$\begin{array}{c} {ExP}_{C}=\frac{C_{\text{PAH}}\times \text{MF}}{B.W.} \end{array}$$

where *C*_PAH_ is the concentration of the type of PAH. All other elements for quantifying the chronic exposures were collected as secondary data. Mass of food (MF) was taken as 33.47 g/day, the total average daily intake of meat and meat products, including offal, derived from the WHO/GEM food cluster diet [21]. While this is considered an overestimation, if the outcome shows little or no risk at such a level, they are valid even more at lower daily intake levels. In the case of carcinogenic congeners, this method is seen on the assumption that a high dose received within a short time is equivalent to consuming a low dose constantly in a lifetime [22]. *B*.*W*. is the average body weight, taken as 60 kg, and considered the average for males and females as recommended by WHO [23].

In characterizing the hazard index (HI) of all PAHs, the hazard quotients (HQs) of the individual PAH congeners were determined by computing their exposure and dividing by their specific reference doses (RFDs) [24], as seen in Equation (2).

(2) $$\begin{array}{c} \text{HQ}=\frac{\text{Exposure dose}}{\text{RFD}} \end{array}$$

Risk assessment was carried out through calculations of margin of exposure (MoE) [22] and excess lifetime cancer risk (ELCR) [22], two quantitative parameters used for risk assessment. The MoE was calculated as a dividend of the *e* benchmark dose lower limit (BMDL_10_) with the exposure as shown in Equation (3) below. The BMDL_10_ is 0.34 mg/kg(bw)/day for *Σ*PAH4, 0.07 mg/kg(bw)/day for BAP, and 0.49 for *Σ*PAH8 according to WHO [24, 25].

(3) $$\begin{array}{c} \text{MoE}=\frac{\text{BMD}{\text{L}}_{10}}{\text{Exposure}} \end{array}$$

BAP, PAH4, and PAH8 were employed as markers of PAH concentrations in the ELCR and MoE models, respectively. The ELCR and MoE calculations were based on Monte Carlo simulation. Before the simulation, the significant variables of the ELCR and MoE models were given an estimated distribution. To guarantee the stability of the results, 10,000 independent iterations of the simulation were done before choosing a random value for each parameter during the Monte Carlo sampling process. According to EFSA, MoE values < 10^4^ are seen as potential causes for concern, 10^4^–10^5^ as a low concern, > 10^5^ as a negligible concern as long as actions are taken to minimize further exposure, and > 106 as negligible concerns. Multiplying the total exposure by the cancer risk value of BAP derived from animal experiments yielded the ELCR values [22, 24] (Equation (4)).

(4) $$\begin{array}{c} \text{ELCR}=7.3\times \text{Exposure} \end{array}$$

If ELCR < 10^−6^, the risk level is deemed tolerable and without adverse effects; interval 10^−6^–10^−4^ values greater than 10^−4^ indicate substantial risk and suggest potential risk.

## 3. Results and Discussion

### 3.1. Concentration of Individual PAH of All 16 Samples From the Four Bushmeat Species

Given the inherent challenges in obtaining substantial quantities of bushmeat, particularly from the target species, the 16 samples representing four distinct species provide a comprehensive overview of bushmeat diversity in this study. While a larger sample size is often desirable, the research design, data collection, and controlled sample handling employed in this investigation mitigate the potential drawbacks of a smaller sample. The exploratory nature of this research, coupled with the heterogeneity of the bushmeat samples, allowed for a focused examination of key variables. Although sample size limitations exist, the depth and quality of the collected data significantly enhance the study's overall robustness.

**Figure 1:**
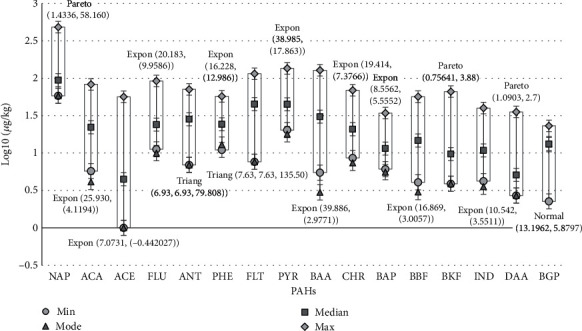
Distributions of PAH in Bushmeat samples (Log_10_ [micrograms per kilogram]).

Figure 1 presents the distributions of PAHs (micrograms per kilogram) in the characterized bushmeat samples. All 16 types of PAHs were found in most of the samples. However, ACE was missing in a number of them.

The amounts of PAH congeners in the bushmeat samples varied from undetectable to 482.68 *μ*g/kg, which was found in naphthalene; Jiang et al., as well as Wahab Abdul, Amoamah, and Abdallah, reported higher concentrations of NAP in smoke bushmeat and other meat products [26, 27]. NAP is a light PAH but can have high toxicity based on the route of exposure [28]. This makes it worrying to have it in higher concentration. In Table 3, the classifications of the International Agency for Research on Cancer (IARC), EFSA, and USEPA show the properties of these PAHs and their sums: *Σ*PAH4 and *Σ*PAH8. BAP concentration ranged from 6.09 to 34.19 *μ*g/kg. The 16 bushmeat samples and total EC-regulated chemical concentrations *Σ*PAH4 group and *Σ*PAH8 group concentrations were also reported. It must be noted that all samples exhibited concentrations of BAP concentration and *Σ*PAH4, *Σ*PAH8, and *Σ*PAH16, substantially higher than those allowed by the E.C. Regulation, indicating that either producers are not concerned about the PAH levels during their production processes or that smoking tends to promote the formation of these compounds [31] highly.

The BAP content for each sample listed in Table 3 is greater than the upper limit established by European Union guidelines for smoked pork [30]. However, the existing research only provides limited information on the PAH contamination levels of smoked bushmeat.

The IARC has classified several PAHs based on their carcinogenicity to humans [15]. BAP is classified as Group 1 (carcinogenic to humans), and BBF is classified as Group 2B (possibly carcinogenic to humans); CHR is classified as Group 3 (not classifiable as to its carcinogenicity to humans), and BKF is classified as Group 3 (not classifiable as to its carcinogenicity to humans) [29].

Similarly, suppose a PAH is classified as Group 2B (possibly carcinogenic to humans). In that case, it means that there is limited evidence suggesting a potential link between exposure and cancer but not enough data for a conclusive determination [15]. The IARC classification is essential for assessing and managing the risks associated with PAH contamination by providing valuable information on their potential carcinogenicity. The classification can help inform risk assessments and regulatory measures to protect human health and the environment. This guides researchers on the potential hazards of exposure to specific PAHs [29].

EFSA provides guidance and recommendations on the maximum levels of certain individual PAHs in food products. EFSA has established maximum levels for BAP, one of the most well-known and highly carcinogenic PAHs [32]. It should be noted that these maximum levels are set as precautionary measures to protect public health and may vary depending on the specific food category. The presence of BAP in PAH4 and PAH8 suggests the level of carcinogenicity of these compounds upon exposure.

Ghana has no explicit regulatory limits on the overall concentration of PAHs in food. The European Union States, however, have limits for smoked meat and smoked meat products that are 2.0 *μ*g for total BAP and 12.0 *μ*g for PAH4 as of 2014 [33]. BAP, the most significant PAH constituent, is regarded as an indicator when measuring the quantity of PAH.

The Ghana Standards Authority, however, refers to the limits established for European Union member states. The nature, source, and type of processing of the meat before eating are called into question by the current findings. The type of processing covers the duration, type, and temperature of the smoking [31]. In brief, when firewood burns incompletely, fat from the meat samples drops into the flames and burns, generating smoke that creates PAHs. The release of PAHs into the environment, from where they can reach foods such as fruit, vegetables, fish, and seafood, of which bushmeat is no exception, is caused by various industrial processes and other anthropogenic factors and natural phenomena.

From Table 4, the average adult population's estimated mean exposure to BAP, *Σ*PAH8, and *Σ*PAH4 from eating bushmeat was calculated. Average BAP concentrations were 14.11, 26.87, and 168.8 *μ*g/kg, respectively, for a total of *Σ*PAH4 and *Σ*PAH8.

### 3.2. Dietary Exposure Estimation

The estimated daily exposure to PAH from bushmeat consumption for adults about BAP, *Σ*PAH4, *Σ*PAH8, and *Σ*PAH16 are presented in Table 5. BAP's exposure ranges from 3.34 at the fifth percentile to 17.39 *μ*g/kg(bw)/day at the 95th percentile. The most frequently occurring (modal) total BAP is 7.87 *μ*g/kg(bw)/day. For *Σ*PAH4, the exposure ranges from 25.11 *μ*g/kg(bw)/day at the 5th percentile to 109.15 *μ*g/kg(bw)/day at the 95th percentile. For *Σ*PAH8, the exposure ranges from 55.76 at the fifth percentile to 228 *μ*g/kg(bw)/day at the 95th percentile, and for *Σ*PAH16, the exposure ranges from 200.35 at the fifth percentile to 639.05 *μ*g/kg(bw)/day at the 95th percentile.

BAP and *Σ*PAH4 dietary exposures from grilled and fried pork products were calculated to be 120 and 74.8 *μ*g/kg(bw)/day, respectively [26]. These were above the daily exposure for smoked bushmeat samples. However, similar research in heat-treated meat doner, chicken doner, meatballs, grilled chicken, and fish samples found estimates of daily intake (EDI) for a total of 16 PAH as 3.41, 3.71, 2.49, 4.12, and 1.77 *μ*g/kg(bw)/day, respectively [34]. These, in contrast, were lower than that of bushmeat. The daily dietary intake of BAP in barbecue, smoked, and dried meat in another study in China ranged from 0.06 to 13.5 *μ*g/kg(bw)/day with a median of 0.69 *μ*g/kg(bw)/day, which was lower than that for the smoked bushmeat in Ghana. PAH from domesticated animals is lower than that of wild animals. This suggests that Ghanaians consume more meat and meat-related products. As a result, individuals who regularly consume significant amounts of meat products may have daily PAH exposure that is significantly higher than the average data [26].

These levels can be avoided if the right equipment and processing conditions are applied. In meat and meat products that have undergone a heat treatment technique known to potentially result in PAH development, it is therefore acceptable to set limit levels for PAH. It has been reported that the amount of PAH in meat products directly correlates with grilling or smoking techniques. Compared to gas and electric oven roasting, charcoal grilling and firewood produced the most PAHs [35]. As the heat source comes into close contact with the meat source, PAHs are more frequently discovered in the outer layer of grilled meat.

Wahab Abdul, Amoamah, and Abdallah [27] quantified the concentration of PAH in bushmeat under different smoking conditions. They stated that smoking with firewood and burnt vehicle tires results in a higher concentration of smoked bushmeat compared to other methods, such as smoking in an oven. As a result of the impact of the carcinogenic endpoints, this result should raise public health concerns. In a similar work done by Santos, Gomes, and Roseiro [36], where traditional Portuguese methods are used to prepare meat and meat products, BAP recorded a maximum level of 7.53 *μ*g/kg(bw)/day and that of CHR as 8.90 *μ*g/kg/day; this is similar to results from this current work with CHR (6.09, 34.19; 5% and 95%). These results were the highest values recorded and were recorded in blood sausages. This was attributed to the presence of internal organs, which are likely to have high-fat content. However, these exposures are far lower than bushmeat smoked using the traditional Ghanaian method.

### 3.3. Health Risk Estimation

From the above table, the MoE ranged from 2.09 × 10^−2^ for BAP, 1.36 × 10^−2^ for *Σ*PAH4, and 1.95 × 10^−2^ for *Σ*PAH8 at 95%, which is hugely lower than 10,000 and thus suggests the probability of a severe health concern to the people who consume smoked bushmeat. These results indicate that at any level of exposure, even at the minimum levels, there will be a higher risk of getting cancer. By extension, the higher cancer risk also results in higher values in the ELTCR values of adult consumers. The risk of getting cancer can be reduced if suitable processing methods reduce the concentration of the contaminants and their associated risk. Dietary exposure is a crucial pathway for humans to be exposed to PAH contaminants. When the MoE approach was used to evaluate the dangers that PAHs posed to Latvian consumers, the MoEs produced ranged from 7205 to 24,434 [37], showing the lower level had higher cancer risk and higher levels of no cancer risk. Sahin et al. [34] revealed that the average MoE value estimated for grilled beef, chicken, and fish was 179.487 and 425.000 for BAP and *Σ*PAH4, which demonstrated a likelihood of increased risk.

The ELCR for BAP ranged from a minimum of 44.77 at 5% to a maximum of 248.53 at 95%. This indicates that at the minor level or minimum level of consumption of smoked bushmeat, there is the highest risk as results are higher compared to food such as cooked rice [28], where the highest figure was recorded at the 95 percentile and was in the 10^−3^ similarly. The obtained values of four groups were all smaller than 10^−4^ when the ELTCR technique was applied to analyze the possible health risks in fried and grilled beef, indicating a tepid potential carcinogenic danger to consumer health [26]. Though our meat consumption and, for that matter, bushmeat are less readily available, the method of processing this kind of meat is standard for all other meat sources: beef, fish, and mutton, even at the home level. The perceived low fat of bushmeat compared to the other domesticated meat sources brings excellent concern to how the risk level of the other meat sources processed in this method is upon consumption since those are readily available. It is conventional to determine the carcinogenicity of every PAH detected in smoked meat products because of how those products are smoked and the environments in which they are exposed to those substances [22]. The ELTCR can be helpful for the health management of the local consumers' intake of processed meat.

## 4. Conclusion

This study reveals alarmingly high levels of carcinogenic PAHs in smoked bushmeat, significantly exceeding EU safety limits. NAP was the predominant PAH, while BAP concentrations were consistently above regulatory thresholds. The risk assessment estimates indicate a substantial health risk to consumers, with MoE and ELCR values falling outside acceptable ranges. These findings underscore the urgent need for stringent regulations governing bushmeat processing and consumption in Ghana to protect public health.

## Figures and Tables

**Table 1:** Analytical quantities of PAH compounds.

**PAHs**	**Symbol**	**Analytical quantities ** **(*μ*g/mL)**
Acenaphthene	ACE	20
Acenaphthylene	ACA	15
Anthracene	ANT	0.8
Benzo[a]anthracene	BAA	4.0
Benzo[b]fluoranthene	BBF	4.0
Benzo[k]fluoranthene	BKF	4.5
Benzo(g,h,i)pyrene	BGP	3.5
Benzo[a]pyrene	BAP	5.0
Chrysene	CHR	3.5
Dibenzo(a,h)anthracene	DAA	3.5
Fluoranthene	FLT	8.0
Fluorene	FLU	5.0
Indeno[1,2,3-cd]pyrene	IND	4.5
Naphthalene	NAP	20.0
Phenanthrene	PHE	3.5
Pyrene	PHY	8.5

**Table 2:** Chromatographic conditions for the extraction of PAH congeners.

GC/MSD	Agilent 7890 GC/Agilent 5975B GC/MS system
Sampler	Agilent 7693 automatic liquid sampler, 5.0 *μ*L syringe (p/n 5181-1273)
PCT device	Purged Ultimate Union (p/n G3186-60580)
Carrier	Helium, constant flow 1.7 mL/min
Restrictor	0.7 m × 0.15 mm id deactivated
PCM 1	3.8 psi constant pressure
MMI	0.5 *μ*L splitless; 320°C, purge flow 50 mL/min at 0.8 min gas saver, 30 mL/min for 2 min

**Table 3:** Concentrations of PAH in smoked bushmeat samples in micrograms per kilogram.

**Sample codes**	**NAP**	**ACA**	**ACE**	**FLU**	**ANT**	**PHE**	**FLT**	**PYR**	**BAA**	**CHR**	**BAP**	**BBF**	**BKF**	**IND**	**DAA**	**BGP**	***Σ*PAH16**	***Σ*PAH8**	***Σ*PAH4**
BS1	482.68	5.74	56.48	17.02	6.93	30.94	7.63	59.47	5.47	14.46	15.44	5.65	4.65	6.66	2.7	2.26	897.32	57.29	42.48
BS2	168.62	40.47	0.00	31.91	27.82	19.38	50.74	77.65	6.97	13.28	8.59	8.89	8.44	19.17	9.1	6.75	497.89	81.19	58.58
BS3	142.62	82.00	0.00	83.22	41.8	18.20	82.02	135.63	7.74	9.20	7.52	4.26	5.65	38.64	32.64	9.16	1395.21	114.81	62.78
BS4	58.16	82.84	0.00	91.89	70.90	14.14	35.34	33.04	9.97	8.59	6.09	4.06	14.43	39.91	35.42	14.61	380.72	133.08	89.64
BS5	102.38	28.07	0.00	19.08	24.31	14.00	38.76	60.00	46.28	27.76	9.81	17.52	16.88	6.55	4.63	17.54	410.18	146.97	79.4
BS6	74.11	26.17	0.00	18.30	26.36	17.29	30.92	39.97	40.95	16.55	9.46	17.52	20.49	5.92	4.55	9.70	790.90	125.14	69.89
BS7	65.99	26.26	0.00	19.05	25.44	15.84	28.20	35.61	28.07	15.83	9.57	15.34	22.04	4.77	5.14	8.60	330.11	109.36	66.18
BS8	64.25	7.62	0.00	22.77	20.43	16.00	20.43	35.18	26.96	11.56	7.57	14.96	24.52	4.21	6.92	6.90	315.12	103.60	54.52
BS9	207.86	45.85	0.00	38.53	49.93	51.12	112.47	115.73	127.84	53.17	34.19	56.74	66.20	17.88	5.56	23.08	645.23	384.66	194.03
BS10	134.69	51.73	0.00	39.47	54.53	53.48	94.33	99.53	96.35	38.59	15.28	36.57	49.21	13.05	5.89	17.07	799.77	272.01	144.97
BS11	91.02	29.14	0.00	22.84	29.65	22.15	52.00	53.77	72.97	30.26	13.59	35.03	49.55	12.38	8.9	14.13	504.58	236.81	108.53
BS12	61.78	9.32	0.00	18.06	13.32	43.61	21.37	20.30	26.31	26.39	12.76	13.96	4.90	10.18	3.19	11.24	1304.35	108.93	66.43
BS13	350.18	48.13	27.22	33.03	54.43	57.30	114.93	102.09	127.76	69.06	25.42	44.04	58.61	18.37	8.95	20.57	1132.87	372.78	192.95
BS14	149.15	9.88	11.51	20.52	24.81	44.46	30.36	29.36	40.00	41.29	22.72	21.41	6.61	14.53	6.02	21.04	477.04	173.62	110.23
BS15	83.79	7.51	8.98	15.54	14.84	32.85	23.77	26.85	35.69	39.60	20.67	23.17	5.70	12.29	5.18	16.67	1609.91	158.97	98.28
BS16	80.54	5.99	8.98	11.22	11.42	32.89	17.88	24.38	26.37	32.48	15.66	15.74	3.88	11.52	3.41	11.82	304.20	120.88	75.3
EFSA	−	−	−	−	−	−	−	−	**+**	**+**	**+**	**+**	**+**	**+**	**+**	**+**			
IARC	2B	−	3	3	3	3	3	3	2B	2B	1	2B	2B	2B	2A	3			
USEPA	**+**	**+**	**+**	**+**	**+**	**+**	**+**	**+**	**+**	**+**	**+**	**+**	**+**	**+**	−	**+**			

*Note:* EFSA [25]. IARC [29]. USEPA [30].

**Table 4:** Elements for the determination of PAH exposure.

**Elements**	**Statistical distribution**	**Statistics**						
		**Min**	**Max**	**Mode**	**Mean**	**Median**	**5%P**	**95%P**
*Σ*PAH16	Loglogistic (16.881, 2.7590)	0.00	482.68	20.51	38.88	9.00	2.55	116.50
*Σ*PAH8	InvGauss (20.496, 17.604)	0.59	127.89	6.09	168.8	21.09	3.97	63.24
*Σ*PAH4	Expon (23.174, 36.979)	3.70	127.84	3.70	26.87	19.76	4.89	73.12
BAP	Expon (8.5562, 5.5552)	5.56	34.19	5.56	14.11	11.49	5.99	31.19
Mass of meat	33.47 kg/day	[21]						
BW_F_	60 kg							
BW_M_	60 kg							

**Table 5:** Exposure of sums of PAHs and their risk indices to smoked meat products.

	**Min**	**Max**	**Median**	**Mean**	**Mode**	**95%**	**5%**
Exposure (*μ*g/kg/day)							
*Σ*PAH16	120	74.17 × 10^6^	284.20	13.13 × 10^2^	258.85	639.05	200.35
*Σ*PAH8	25.55	11.37 × 10^6^	96.10	499.04	83.83	238.68	55.76
*Σ*PAH4	11.12	315.40	52.28	57.81	43.04	109.15	25.11
Total BAP	3.09	70.49	6.407	7.87	3.12	17.39	3.34
Risk indices							
Hazard index	1.09 × 10^−4^	4.5	3.04 × 10^−4^	1.6 × 10^−3^	3.04 × 10^−4^	8.23 × 10^−4^	2.061 × 10^−4^
Margin of exposure							
MoE (BAP)	1.16 × 10^−3^	2.26 × 10^−2^	1.09 × 10^−2^	1.15 × 10^−2^	7.2 × 10^−3^	2.09 × 10^−2^	4.02 × 10^−3^
MoE (*Σ*PAH4)	9.31 × 10^−4^	2.78 × 10^−2^	6.49 × 10^−3^	7.15 × 10^−3^	6.49 × 10^−3^	1.36 × 10^−2^	3.66 × 10^−3^
MoE (PAH8)	1.59 × 10^−3^	4.35 × 10^−2^	9.37 × 10^−3^	1.03 × 10^−2^	8.58 × 10^−3^	1.95 × 10^−2^	4.49 × 10^−3^
Excess lifetime cancer risk							
ELTCR_(BAP)_	44.27	883.01	91.53	112.45	44.61	248.53	47.77

## Data Availability

Data will be available on request to the authors. However, all relevant data relating to the work are part of the content of the manuscript.
